# Evolutionary Trajectory of Radioresistance in Lung Cancer Brain Metastasis Organoids

**DOI:** 10.1186/s12575-026-00339-z

**Published:** 2026-04-21

**Authors:** Jianing Chen, Cize Gao, Li Wang, Leilei Wu, Boyue Pang, Chunxia Su

**Affiliations:** 1 Department of Comprehensive Oncology Center, Shanghai Pulmonary Hospital & Thoracic Cancer Institute, Tongji University School of Medicine, Shanghai, China; 2 Department of Radiation Oncology, Tongji University School of Medicine, Shanghai, China

**Keywords:** Lung Cancer Brain Metastasis, Organoids, Radioresistance, Single-Cell RNA Sequencing, Metabolic Reprogramming

## Abstract

**Background:**

Radiotherapy (RT) is a cornerstone treatment for lung cancer brain metastasis (LCBM), yet acquired radioresistance frequently leads to recurrence. The molecular and metabolic mechanisms underlying this adaptive evolution at single-cell resolution remain poorly defined.

**Methods:**

Patient-derived organoids (PDOs) from LCBM tissues were established to model clinical radiation responses. Paired pre- and post-RT samples underwent single-cell RNA sequencing (scRNA-seq) to delineate transcriptional, metabolic, and regulatory alterations associated with radioresistance.

**Results:**

Single-cell analysis revealed substantial population remodeling following RT, characterized by depletion of proliferative cells and enrichment of a resilient Hypoxic-EMT subpopulation. Pseudotime analysis demonstrated lineage plasticity, showing a transition from proliferative to mesenchymal states. Mechanistically, a viral mimicry response involving NF-κB and STAT signaling supported stress adaptation. Resistant cells exhibited a hypermetabolic phenotype marked by metabolic plasticity, including hybrid bioenergetics coupling glycolysis with oxidative phosphorylation, enhanced lipid turnover via simultaneous fatty acid synthesis and degradation, and increased glutathione metabolism for reactive oxygen species buffering. Pharmacogenomic profiling indicated concurrent chemotherapy resistance but collateral sensitivity to PI3K/MEK inhibitors and epigenetic therapies.

**Conclusions:**

These findings provide a high-resolution atlas of radioresistance in LCBM and suggest that targeting the Hypoxic-EMT niche or oxidative-antioxidant balance may overcome therapeutic resistance.

## Introduction

Lung cancer remains the leading cause of cancer-related mortality worldwide, with non-small cell lung cancer (NSCLC) accounting for approximately 85% of cases [1]. Brain metastasis (BM) represents one of the most devastating complications of NSCLC, affecting 20% to 40% of patients during the course of their disease [2]. Despite recent advances in targeted therapies and immunotherapy, the prognosis for patients with LCBM remains dismal, with a median overall survival of less than 12 months [3]. Radiotherapy (RT) serves as a cornerstone treatment for local control; however, the development of acquired radioresistance inevitably leads to tumor recurrence and therapeutic failure [4]. Therefore, elucidating the molecular mechanisms underlying radioresistance is urgently needed to improve clinical outcomes.

To investigate resistance mechanisms, appropriate preclinical models are essential. While traditional two-dimensional (2D) cell lines have been widely used, they often fail to recapitulate the complex histological architecture and genomic heterogeneity of the parental tumors [5]. Patient-derived xenograft (PDX) models preserve these features but are limited by long establishment times and low throughput [6]. In contrast, patient-derived organoids (PDOs) have emerged as a superior ex vivo model system. PDOs faithfully retain the histopathological and genetic characteristics of the original patient tumors while allowing for efficient, time-course manipulation of therapeutic interventions [7, 8]. Thus, PDOs serve as ideal “patient avatars” for modeling dynamic responses to radiotherapy in a controlled environment.

Tumors are inherently heterogeneous ecosystems composed of diverse cell subpopulations with distinct sensitivities to treatment [9]. Traditional bulk RNA sequencing, which averages gene expression across the entire tissue, often masks rare but critical subpopulations that drive therapeutic resistance [10]. Single-cell RNA sequencing (scRNA-seq) has revolutionized cancer research by offering cellular-level resolution. This technology enables the dissection of intratumoral heterogeneity, the identification of rare resistant subclones, and the reconstruction of developmental trajectories under therapeutic pressure [11–13]. By leveraging scRNA-seq, researchers can trace the evolutionary path of tumor cells as they adapt to stress, revealing transcriptional programs that are invisible to bulk analysis.

In this study, we integrated patient-derived organoid culture with time-series single-cell transcriptomics to map the evolutionary landscape of radioresistance in NSCLC brain metastasis. By comparing the transcriptomic profiles of tumor cells before and after radiation, we aimed to dissect the cellular heterogeneity and identify the specific subpopulations responsible for therapeutic failure. Specifically, we sought to determine whether resistance arises from the selection of pre-existing stem-like clones or through transcriptional reprogramming toward an adaptive state. Our findings uncover a dynamic transition toward a hypoxic-dormant phenotype driven by specific transcription factor networks, providing novel insights and potential therapeutic targets to overcome radioresistance in lung cancer brain metastasis.

## Materials and Methods

### Generation and Radiotherapy of Lung Cancer Brain Metastasis Organoids

Tumor tissue samples were obtained from patients with lung cancer brain metastasis (LCBM) under a protocol approved by the Ethics Committee of Shanghai Pulmonary Hospital. To establish organoids, fresh tumor tissues were collected and transported in pre-cooled preservation solution at 4 °C, and processed within 24 h. After washing with PBS and surface sterilization with 75% ethanol for 10 s, the tissues were rinsed and minced into fragments of approximately 1 mm³. The tissue fragments were dissociated using a tissue digestion solution on a shaker at 37 °C for 40 min. The digestion process was monitored microscopically and terminated with human AB serum upon the release of single cells. The suspension was filtered through a 70-µm cell strainer, followed by centrifugation at 1500 rpm for 5 min at 4 °C. Red blood cells were removed using a lysis buffer when necessary.

For organoid culture, cell viability and number were quantified using AO/PI staining. The cell pellet was resuspended in Matrigel (Corning) at a density of approximately 1 × 10⁵ cells per 10 µL. The suspension was seeded as 10-µL domes into 48-well plates and inverted for 10 min to ensure uniform cell distribution during solidification. After the Matrigel hardened, specific lung cancer organoid culture medium was added. The cultures were maintained at 37 °C in a humidified atmosphere containing 5% CO₂, with the medium replenished every 2–3 days.

### Single-cell RNA Sequencing Library Preparation and Data Preprocessing

Single-cell suspensions were processed into single-cell RNA-seq libraries using a commercially available high-throughput single-cell sequencing platform following the manufacturer’s standard instructions. Sequencing was performed on a high-throughput sequencing system. Raw sequencing reads were aligned to the human reference genome (GRCh38) and quantified to generate a gene expression matrix using a standard upstream bioinformatics pipeline. The resulting data were subsequently processed using the Seurat R package (v5.0) [14]. Stringent quality control was applied: cells were retained if they had (1) gene counts between 200 and 6,000; and (2) mitochondrial gene percentage less than 15%. Doublets were predicted and removed using DoubletFinder [15]. Data were log-normalized (NormalizeData), and the top 2,000 highly variable genes (HVGs) were identified (FindVariableFeatures, method = “vst”). To mitigate batch effects between pre- and post-radiotherapy samples, the Harmony algorithm was employed for data integration [16]. Dimensionality reduction was performed via Principal Component Analysis (PCA), and the top 30 PCs were utilized for clustering (FindNeighbors, FindClusters, resolution = 0.5) and visualization using Uniform Manifold Approximation and Projection (UMAP) [17, 18].

### Cell Type Annotation and Copy Number Variation Analysis

Cell clusters were annotated based on the expression of canonical marker genes: Epithelial/Tumor cells (*EPCAM*,* KRT18*,* KRT19*), T cells (*CD3D*,* CD3E*), Myeloid cells (*CD68*,* LYZ*), B cells (*CD79A*,* MS4A1*), and Fibroblasts (*COL1A1*,* DCN*). To distinguish malignant cells from non-malignant stromal cells, large-scale Copy Number Variation (CNV) analysis was performed using the inferCNV R package (v1.12.0; https://github.com/broadinstitute/inferCNV). T cells and myeloid cells served as the genomic reference baseline. The analysis utilized a Hidden Markov Model (HMM) (denoise=TRUE, HMM=TRUE, cutoff = 0.1) [19, 20]. A CNV score was calculated for each cell to quantify genomic instability, defined as the mean squared deviation from the reference baseline across all genomic windows.

### Differential Expression and Functional Module Scoring

Differentially expressed genes (DEGs) between radiotherapy-treated (4 Gy) and untreated (0 Gy) groups were identified using the Wilcoxon rank-sum test (FindMarkers). Significance thresholds were set at adjusted *p* value < 0.05 and |log_2_FC| > 0.25. Functional enrichment analyses (GO and KEGG) were conducted using the clusterProfiler package.

### Transcription Factor (TF) Regulatory Network Analysis

TF activity was inferred from gene expression data using the DoRothEA (Discriminant Regulon Expression Analysis) package coupled with the decoupleR algorithm [21, 22]. Only regulons with high confidence (confidence levels A, B, and C) were included. The weighted mean method (run_wmean) was used to compute single-cell TF activity scores. Differential TF activity analysis was performed to identify key regulators driving the response to radiotherapy. Functional enrichment (GO/KEGG) of the target genes regulated by these differential TFs was conducted to elucidate their biological functions. Furthermore, TF-target regulatory networks were constructed and visualized to highlight master regulators in the post-radiation context.

### Pseudotime Trajectory Analysis

To reconstruct the developmental trajectory of tumor cells under radiotherapy pressure, pseudotime analysis was performed using Monocle 2 [23]. The raw count matrix of epithelial cells was converted into a CellDataSet object. Ordering genes were selected based on differential expression between clusters (q-value < 0.01). The trajectory was constructed using the DDRTree algorithm. Cells were ordered along the pseudotime axis to delineate the transition from the “Sensitive State” (early pseudotime) to the “Resistant State” (late pseudotime), validated by the distribution of 0 Gy and 4 Gy cells.

### Single-cell Metabolic Profiling

Metabolic heterogeneity was assessed using the scMetabolism R package. This method quantified the activity of metabolic pathways at the single-cell level based on the KEGG databases using the VISION algorithm. The average metabolic scores were calculated for pre- and post-radiotherapy groups, as well as for different sub-clusters. Differential metabolic pathways were identified and visualized using heatmaps and dot plots to reveal metabolic reprogramming induced by radiotherapy.

### Drug Sensitivity Prediction

To predict clinical drug response, we utilized the oncopredict R package [24]. The gene expression profiles from the Genomics of Drug Sensitivity in Cancer (GDSC2) database were used as the training set [25]. A Ridge Regression model was constructed to predict the half-maximal inhibitory concentration (IC50) of chemotherapeutic and targeted drugs for each single cell in our dataset. Lower predicted IC50 values indicated higher drug sensitivity. We compared the estimated drug sensitivities between pre- and post-RT groups and across sub-clusters to identify potential therapeutic agents capable of overcoming radiation resistance.

### Statistical Analysis

Statistical analyses were performed using GraphPad Prism (version 10.0.3) and R software (version 4.1.2). The data shown (unless otherwise indicated) represented results from at least three independent experiments. Data were expressed as the mean ± standard deviation (SD) or standard error of the mean (SEM). Statistical significance between two groups was determined using two-sided Student’s t-test or Wilcoxon rank-sum test, depending on the data distribution. *P*-value < 0.05 was considered statistically significant.

## Results

### scRNA-seq Reveals Functional Heterogeneity and Radiotherapy-Induced Population Remodeling in LCBM Organoids

**Fig. 1:**
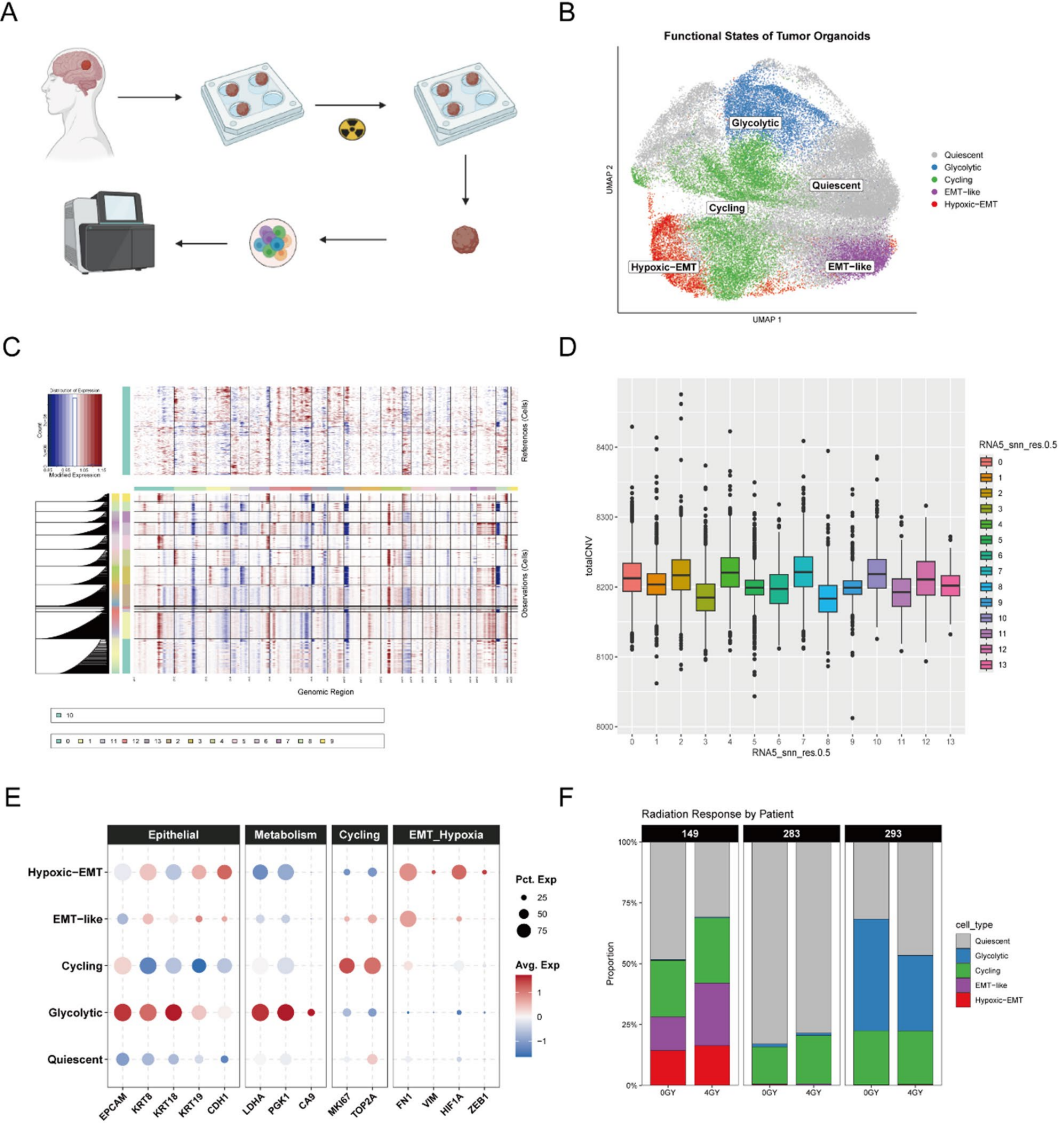
Single-cell transcriptomic atlas of radiotherapy-treated LCBM organoids. **A** Schematic workflow of the study. Patient-derived LCBM organoids were cultured, treated with radiotherapy (0 Gy vs. 4 Gy), and dissociated for single-cell RNA sequencing (scRNA-seq) to profile transcriptional heterogeneity. **B** UMAP projection of single cells from all samples, revealing five distinct functional subpopulations: Quiescent (Grey), Glycolytic (Blue), Cycling (Green), EMT-like (Purple), and Hypoxic-EMT (Red). **C** Heatmap of inferred chromosomal Copy Number Variations (CNVs) using inferCNV. Red indicates amplifications and blue indicates deletions. **D** Box plot showing the distribution of total CNV scores across different clusters. The consistently high scores confirm the malignant identity of all identified subpopulations. **E** Dot plot visualizing the expression of canonical marker genes defining cell identities. Dot size represents the percentage of expressing cells, and color intensity indicates average expression levels. The clusters are annotated based on Epithelial (*EPCAM*,* CDH1*), Metabolism (*LDHA*,* PGK1*), Cycling (*MKI67*), and EMT/Hypoxia (*FN1*,* VIM*,* HIF1A*) signatures. **F** Stacked bar plots showing the proportional distribution of cell subpopulations in Pre-RT (0 Gy) and Post-RT (4 Gy) groups across three biological replicates (149, 283, 293)

To investigate the cellular ecosystem and radiation response mechanisms in LCBM, we established PDOs and subjected them to radiotherapy (4 Gy) or control treatment (0 Gy), followed by high-throughput single-cell RNA sequencing (Fig. 1A). After stringent quality control and dimensionality reduction, unsupervised clustering identified five distinct tumor subpopulations based on their transcriptomic signatures (Fig. 1B). To distinguish malignant cells from potential non-tumor contaminants, we performed Copy Number Variation (CNV) inference (Fig. 1C). The analysis revealed widespread chromosomal alterations across all identified clusters compared to the reference baseline. Furthermore, quantitative scoring showed consistently high CNV burdens across all clusters with no significant differences (Fig. 1D), confirming that the organoids consist predominantly malignant cells and that the identified heterogeneity reflects intrinsic functional states rather than genetic subclones.

We next annotated these functional states based on canonical phenotypic markers (Fig. 1E). The Cycling cluster was characterized by high expression of proliferation markers (*MKI67*,* TOP2A*). The Glycolytic and Quiescent clusters maintained a classic epithelial phenotype (*EPCAM*^*+*^, *CDH1*^*+*^) with the former enriched in glycolysis-associated genes (*LDHA*,* PGK1*). In contrast, the EMT-like and Hypoxic-EMT clusters displayed a clear loss of epithelial features and a gain of mesenchymal traits (*FN1*^*+*^, *VIM*^*+*^), with the latter specifically co-expressing hypoxia-response genes (*HIF1A*,* ZEB1*).

Crucially, comparative analysis of cell proportions revealed a dramatic population shift post-radiotherapy (Fig. 1F). Across three biological replicates, the 4 Gy treatment led to a marked reduction in the Glycolytic and Cycling fractions, accompanied by a substantial expansion of the Hypoxic-EMT and EMT-like subpopulations. This distinct remodeling suggests that radiotherapy selectively eliminates rapidly dividing and metabolically active epithelial cells, while enriching for stress-resistant populations with mesenchymal and hypoxic features.

### Radiotherapy Triggers a Robust Antiviral and DNA Damage Response Without Compromising Proliferation Potential

**Fig. 2:**
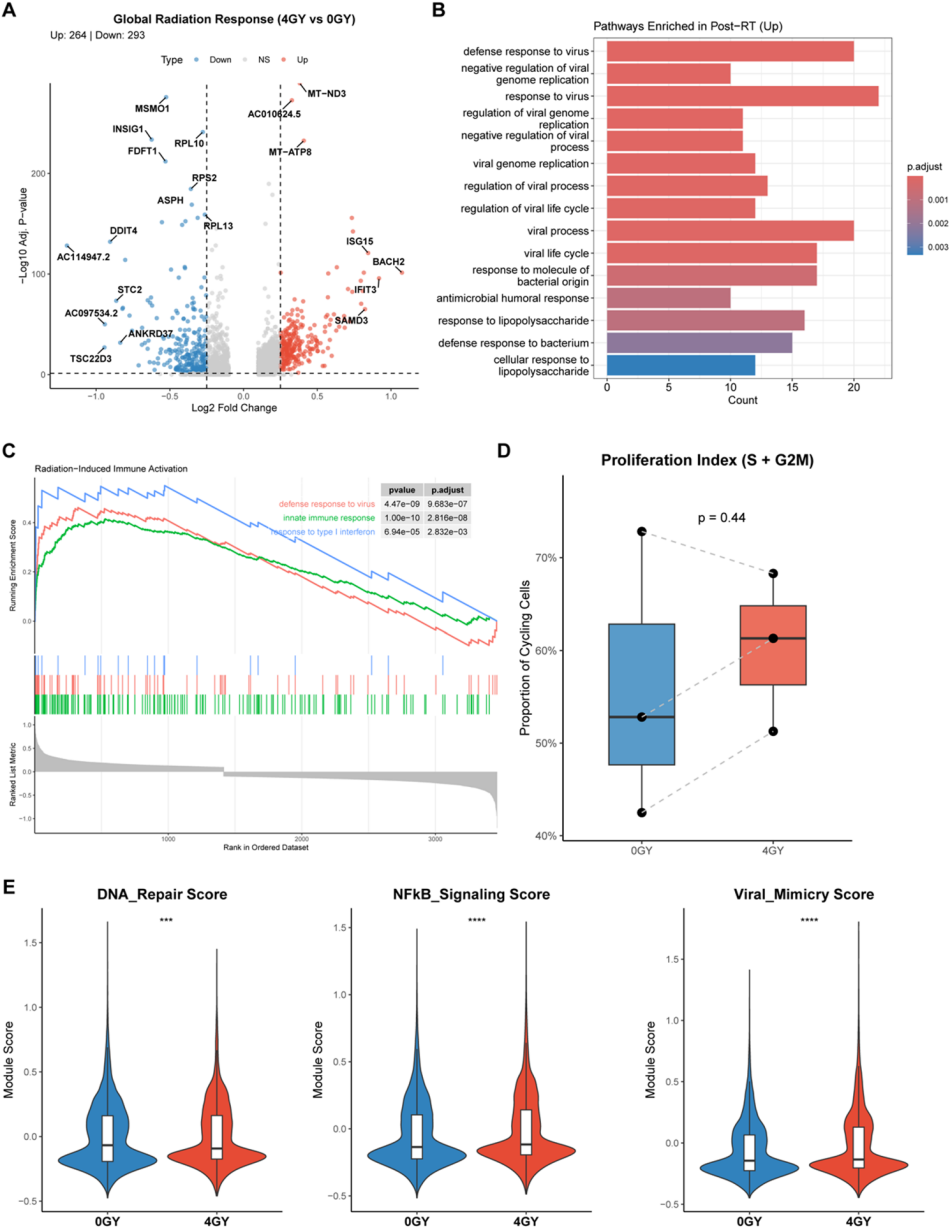
Transcriptional landscape of radiotherapy response. **A** Volcano plot illustrating the differentially expressed genes (DEGs) between the Post-RT (4 Gy) and Pre-RT (0 Gy) groups. Red dots represent upregulated genes (*n* = 284), and blue dots represent downregulated genes (*n* = 293). Key genes are labeled. Vertical dashed lines indicate a |log_2_FC| cutoff of > 0.25, and the horizontal line indicates an adjusted p-value < 0.05. **B** Bar plot showing the top enriched biological processes (GO/KEGG) for the genes upregulated in the Post-RT group. The length of the bar represents the gene count, and the color intensity represents the statistical significance (adjusted p-value). The results highlight a strong enrichment in viral defense and immune response pathways. **C** Gene Set Enrichment Analysis (GSEA) plots demonstrating the significant enrichment of “Defense Response to Virus,” “Innate Immune Response,” and “Response to Type I Interferon” gene sets in the Post-RT group. **D** Box plot comparing the Proliferation Index (proportion of cells in S and G2/M phases) between Pre-RT (0 Gy) and Post-RT (4 Gy) groups. No statistically significant difference was observed (*p* = 0.44, Wilcoxon rank-sum test), indicating sustained proliferation potential post-radiation. **E** Violin plots showing the distribution of module scores for DNA Repair, NF-κB Signaling, and Viral Mimicry in single cells from Pre-RT (Blue) and Post-RT (Red) groups. The Post-RT group exhibits significantly higher activity in all three pathways (***p < 0.001, ****p < 0.0001; Wilcoxon rank-sum test). The white box inside the violin represents the interquartile range

To dissect the molecular mechanisms underlying the radiation response in LCBM organoids, we performed differential expression analysis between the radiotherapy-treated (4 Gy) and untreated (0 Gy) groups. This comparison identified a total of 577 differentially expressed genes (DEGs), with 284 upregulated and 293 downregulated genes in the post-radiation group (Fig. 2A).

Functional enrichment analysis of the upregulated genes revealed a striking activation of immune-related pathways. KEGG pathway analysis (Fig. 2B) showed a significant enrichment in “defense response to virus,” “viral genome replication,” and “response to type I interferon.” Consistent with this, Gene Set Enrichment Analysis (GSEA) (Fig. 2C) demonstrated a robust positive enrichment for “Response to Virus” and “Innate Immune Response” signatures in the post-radiation group. This suggests that radiotherapy induces a state of “viral mimicry,” likely through the sensing of cytosolic DNA released from radiation-induced damage, which in turn activates innate immune signaling.

To further quantify these functional alterations, we calculated specific module scores for key biological processes. The post-radiation group exhibited significantly higher scores for DNA Repair, NF-κB Signaling, and Viral Mimicry compared to the control group (Fig. 2E), confirming the activation of stress response and inflammatory pathways. Interestingly, despite the significant DNA damage response, there was no significant difference in the proliferation index (S phase and G2/M phase cells) between the 0 Gy and 4 Gy groups (*p* = 0.44) (Fig. 2D). This indicates that a subpopulation of cells successfully bypasses radiation-induced cell cycle arrest and maintains their proliferative capacity, potentially serving as the seed for recurrence.

Collectively, these transcriptomic signatures portray a scenario where surviving tumor cells adapt to radiotherapy by upregulating DNA repair and inflammatory survival signals (NF-κB) while sustaining their growth potential, highlighting the resilience of LCBM organoids.

### Radiotherapy Induces a Transcriptional Switch Driven by NF-κB and STAT Regulatory Networks

**Fig. 3:**
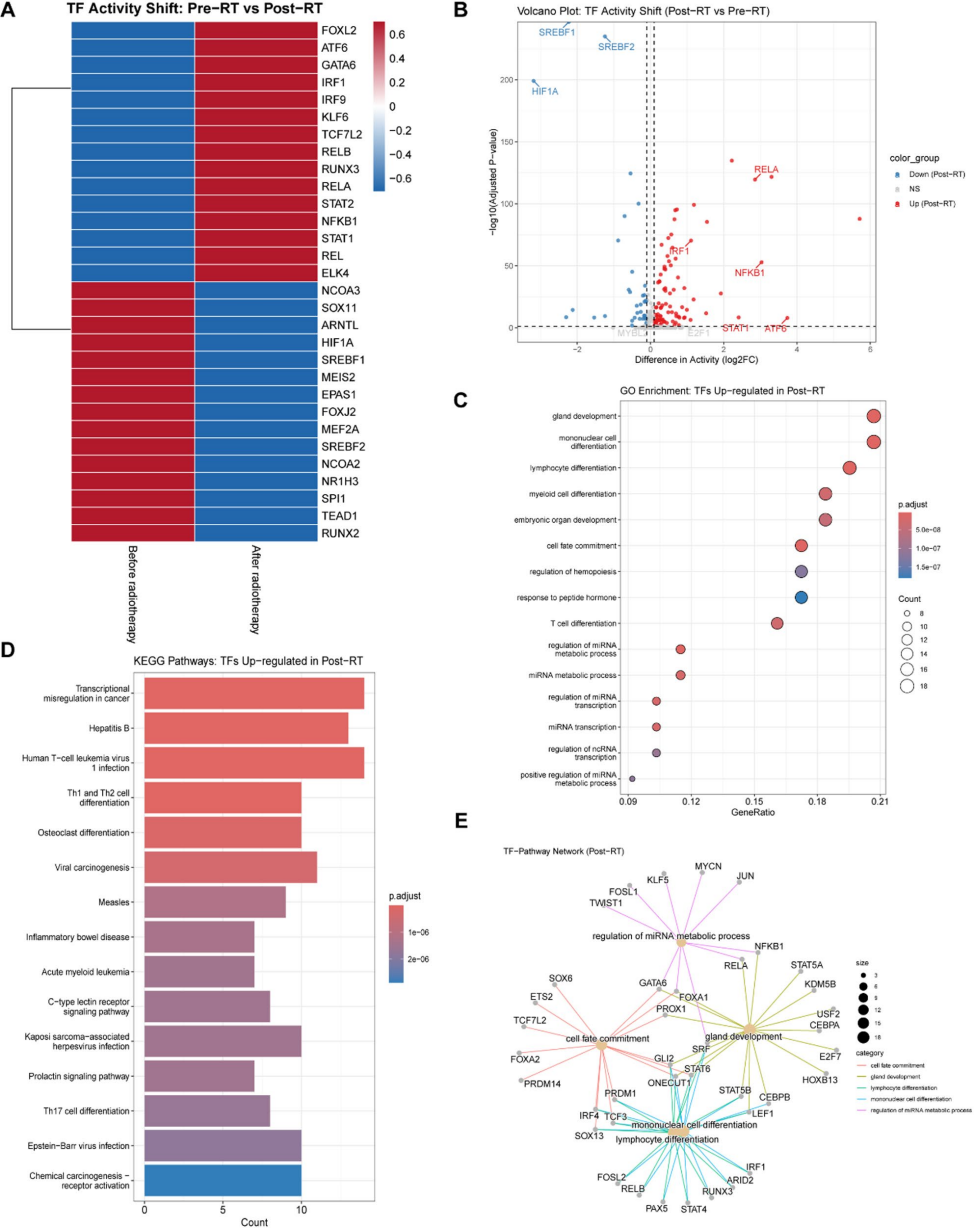
Identification of master regulators driving radiotherapy response. **A** Heatmap showing the differential activity of the top variable transcription factors (TFs) between Before Radiotherapy (0 Gy) and After Radiotherapy (4 Gy) groups. Activity scores were inferred using DoRothEA. Red indicates high activity; Blue indicates low activity. **B** Volcano plot displaying the differential TF activity between Post-RT and Pre-RT groups. Red dots represent significantly upregulated TFs (e.g., *RELA*,* NFKB1*,* STAT1*) in the Post-RT group, while blue dots represent downregulated TFs (e.g., *SREBF1*,* SREBF2*). Vertical dashed lines indicate |log_2_FC| > 0.5. **C** Gene Ontology (GO) enrichment analysis of the TFs upregulated in the Post-RT group. Dot size represents the gene count, and color indicates the adjusted p-value. **D** KEGG pathway enrichment analysis of the TFs upregulated in the Post-RT group, highlighting inflammatory and cancer-related pathways. **E** TF-Pathway regulatory network constructed for the Post-RT group. The network visualizes the connections between key upregulated TFs (e.g., *RELA*,* NFKB1*) and their downstream biological processes (e.g., cell fate commitment), demonstrating the regulatory logic of radioresistance

To identify the master regulators orchestrating the transcriptional reprogramming and acquired resistance post-radiotherapy, we inferred transcription factor (TF) activities using the DoRothEA algorithm. Comparative analysis of TF activity scores between Pre-RT and Post-RT groups revealed a profound shift in the regulatory landscape (Fig. 3A).

The differential activity analysis highlighted a distinct set of TFs activated by radiotherapy (Fig. 3B). Most notably, we observed a robust upregulation of the NF-κB family (e.g., *RELA*,* NFKB1*,* REL*) and STAT family (e.g., *STAT1*,* STAT2*) in the Post-RT group. This suggests that surviving cells rely on inflammatory and interferon-related signaling for survival. Conversely, TFs regulating lipid biosynthesis, such as *SREBF1* and *SREBF2*, were significantly downregulated.

Functional enrichment analysis of the upregulated TFs provided further insight into their biological roles. GO annotation (Fig. 3C) indicated that these master regulators are primarily involved in “cell fate commitment” and “myeloid/lymphocyte differentiation,” suggesting a dedifferentiation or lineage plasticity process. KEGG pathway analysis (Fig. 3D) confirmed the enrichment of “Transcriptional misregulation in cancer” and “Viral carcinogenesis” pathways, pointing towards a stress-induced “viral mimicry” state or chronic inflammation.

To visualize the regulatory hierarchy, we constructed a TF-target network for the Post-RT group (Fig. 3E). The network topology placed *RELA*,* NFKB1*, and *STAT1* at the central hubs, densely connected to gene modules driving “cell fate commitment” and “gland development.” This indicates that the NF-κB/STAT axis acts as the core engine driving the post-radiation adaptive response and phenotypic plasticity.

### Pseudotime Trajectory Analysis Reconstructs the Evolution

To delineate the dynamic lineage plasticity driving radioresistance, we reconstructed the single-cell trajectory using Monocle 2. The analysis projected cells onto a continuous pseudotime axis, revealing a bifurcating structure that reflects the evolutionary history of the tumor organoids under stress.

**Fig. 4:**
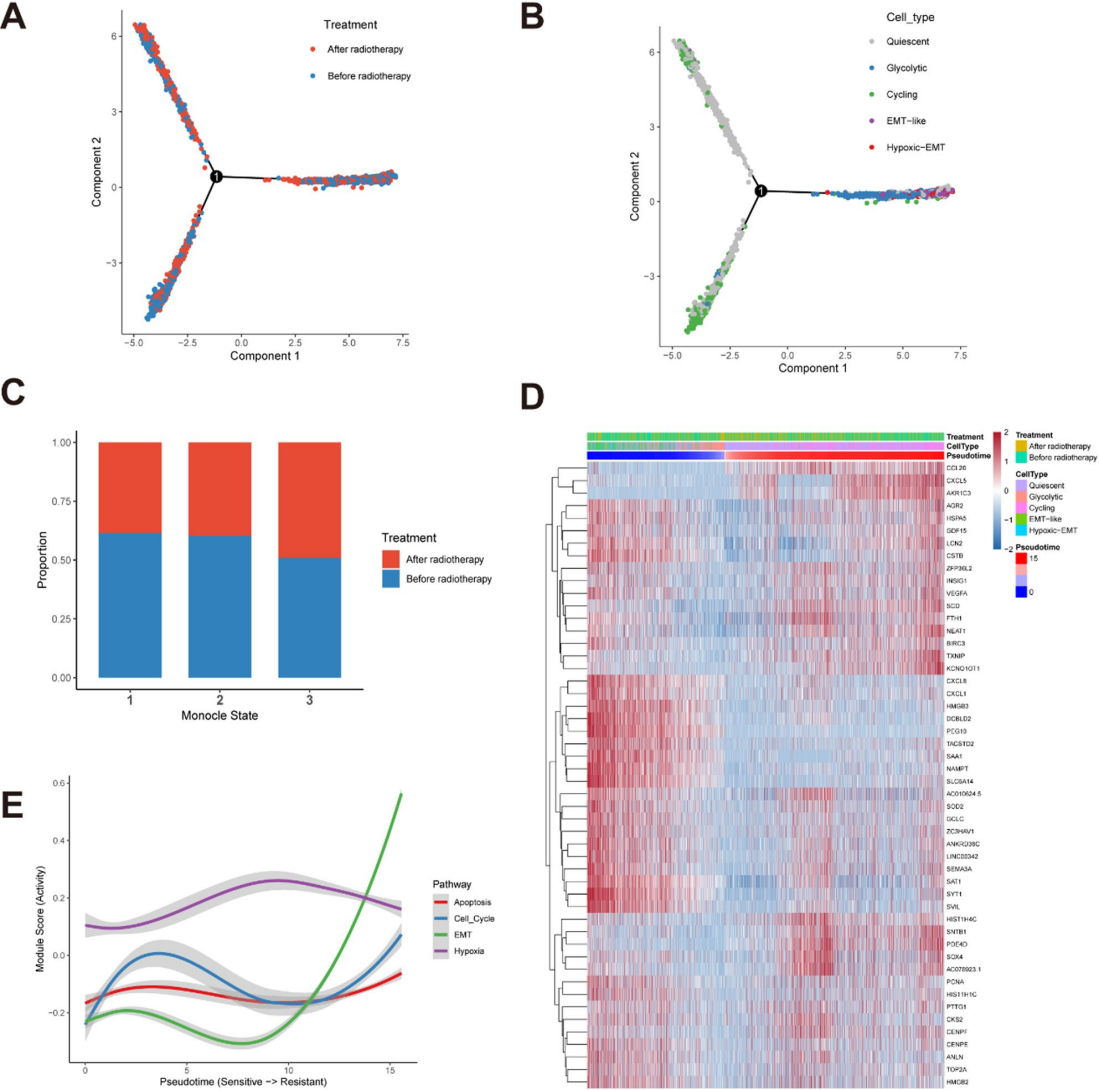
Pseudotime trajectory analysis and transcriptional dynamics. **A** The single-cell trajectory constructed by Monocle 2, colored by treatment groups. The plot reveals a directional progression from Pre-RT cells (Blue, concentrated at the root) to Post-RT cells (Red, concentrated at the terminus). **B** The same trajectory colored by cell subpopulations. The evolutionary path spans from a proliferative start point (Cycling/Glycolytic) to a resistant endpoint (Hypoxic-EMT/EMT-like). **C** Stacked bar plot showing the proportional distribution of Pre-RT and Post-RT cells across the three identified Monocle states, confirming the enrichment of treated cells in the terminal state. **D** Heatmap of differentially expressed genes varying as a function of pseudotime. Rows represent genes and columns represent single cells ordered from the root to the terminus. Key markers (e.g., MKI67, FN1) mark the transition from proliferation to mesenchymal traits. The color scale indicates relative gene expression levels (Red: High; Blue: Low). **E** Line plot illustrating the dynamic changes in pathway activity scores (Cell Cycle, EMT, Hypoxia, Apoptosis) along the pseudotime axis. The curves highlight a functional switch from proliferation to a hypoxic-mesenchymal state: Hypoxia activation precedes the EMT surge. Shaded areas represent the confidence interval

We first examined the global distribution of treatment groups along this trajectory (Fig. 4A). Pre-RT cells were predominantly located at the root and early branches, whereas Post-RT cells were heavily enriched at the distal terminus of the trajectory. This distinct separation indicates that radiotherapy drives a directional selection process. By mapping the cell subpopulations onto the same trajectory (Fig. 4B), we resolved the biological identity of this evolution: the root consisted mainly of Quiescent and Cycling cells, while the radiation-enriched terminus was almost exclusively occupied by the Hypoxic-EMT and EMT-like subpopulations. Quantitative analysis of the composition of the three branches confirmed this shift, showing a dramatic accumulation of Post-RT cells in the terminal state (State 3) compared to the diverse distribution of Pre-RT cells (Fig. 4C).

To uncover the molecular logic governing this transition, we analyzed the transcriptomic dynamics along the pseudotime axis. The global heatmap (Fig. 4D) displayed a clear cascade of gene expression changes. We further quantified this functional switch by analyzing pathway activity scores (Fig. 4E). The analysis revealed a specific temporal sequence: “Hypoxia” signaling was activated early and sustained, potentially serving as the initial stress response. Conversely, “EMT” pathway activity remained suppressed initially but exhibited an exponential surge at the late stage, indicating that EMT is a terminal event in acquiring resistance. Simultaneously, “Cell Cycle” and “Apoptosis” activities declined, confirming that radiotherapy forces tumor cells to exit the cell cycle and acquire a non-proliferative, hypoxic-mesenchymal phenotype.

### Radiotherapy Induces Metabolic Quiescence with Selective Reliance on Oxidative Phosphorylation and ROS Defense

**Fig. 5:**
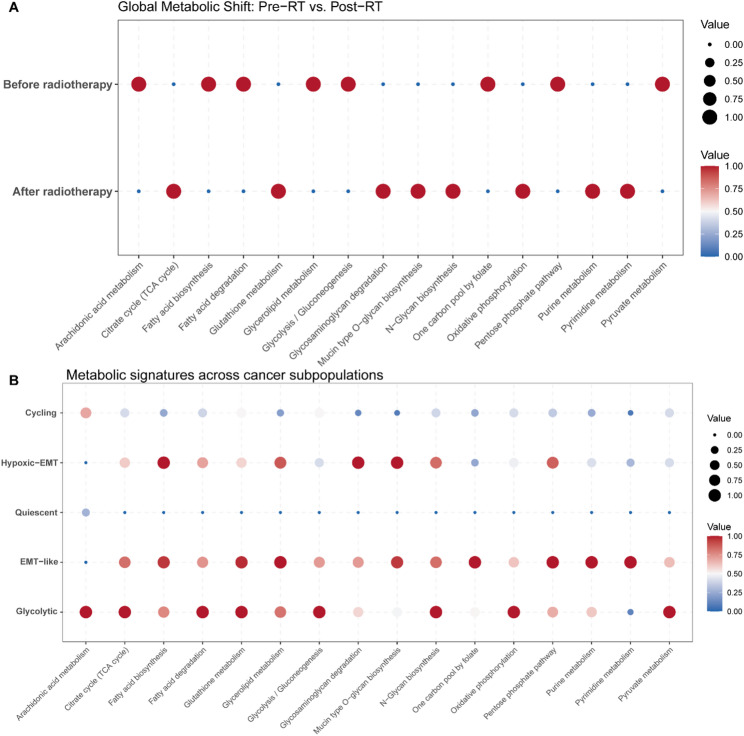
Single-cell metabolic landscape and reprogramming. **A** Dot plot illustrating the global metabolic shift between Before Radiotherapy (0 Gy) and After Radiotherapy (4 Gy) samples. The size and color of the dots represent the average metabolic pathway activity scores. The Pre-RT group is characterized by a “hyper-metabolic” state with high activity in glycolysis, fatty acid biosynthesis, and fatty acid degradation. In contrast, the Post-RT group exhibits “metabolic quiescence” marked by the downregulation of lipid and sugar metabolism, yet displays selective upregulation of survival pathways, including the TCA cycle, oxidative phosphorylation, glutathione metabolism, and glycan/mucin biosynthesis. **B** Dot plot showing metabolic heterogeneity across subpopulations. Unlike the metabolically inactive Quiescent cluster, the Hypoxic-EMT subpopulation (bottom row) exhibits a distinct hyper-metabolic phenotype. It is characterized by the concurrent upregulation of central carbon metabolism (Glycolysis, TCA cycle, OXPHOS), active lipid turnover (Fatty acid biosynthesis and degradation), and robust stress defense (Glutathione metabolism), indicating a highly plastic and adaptive metabolic strategy that sustains radioresistance

To dissect the metabolic alterations underlying radioresistance, we quantified pathway activities at single-cell resolution using scMetabolism. Comparing the global metabolic landscapes revealed a dramatic transition from a hyper-metabolic state to a specific survival mode post-radiotherapy (Fig. 5A). Prior to treatment (Pre-RT), tumor cells exhibited robust activity across a broad spectrum of metabolic pathways, including Glycolysis/Gluconeogenesis, Fatty acid biosynthesis, and Fatty acid degradation. This profile reflects the high bioenergetic and biosynthetic demands of rapidly proliferating tumors, engaging in both lipid synthesis and turnover. In stark contrast, the residual cells surviving radiotherapy (Post-RT) displayed a global suppression of these anabolic pathways. Both Fatty acid biosynthesis and degradation were significantly downregulated, indicating a cessation of lipid turnover consistent with cell cycle arrest. However, amidst this broad metabolic quiescence, specific survival pathways were selectively upregulated. The Post-RT group showed enriched activity in the Citrate cycle (TCA), Oxidative phosphorylation (OXPHOS), and Glutathione metabolism.

Subpopulation analysis clarified the cellular source of this signature (Fig. 5B). The radiation-sensitive Cycling and Glycolytic clusters were the primary contributors to the high lipid/glycolytic metabolism observed Pre-RT. Conversely, the resistant populations exhibited distinct survival strategies. The EMT-like subpopulation emerged as the metabolic “powerhouse” of resistance, displaying low lipid metabolism but high levels of Oxidative phosphorylation and Glutathione metabolism. Meanwhile, the Hypoxic-EMT subpopulation appeared largely metabolically quiescent, further supporting a dormancy-like state. Collectively, these data suggest that radioresistance is driven by a switch from biomass accumulation (lipids) to highly efficient mitochondrial energy production and antioxidant defense.

### Pharmacogenomic Profiling Reveals Radiotherapy-Induced Drug Resistance and Emerging Therapeutic Vulnerabilities

**Fig. 6:**
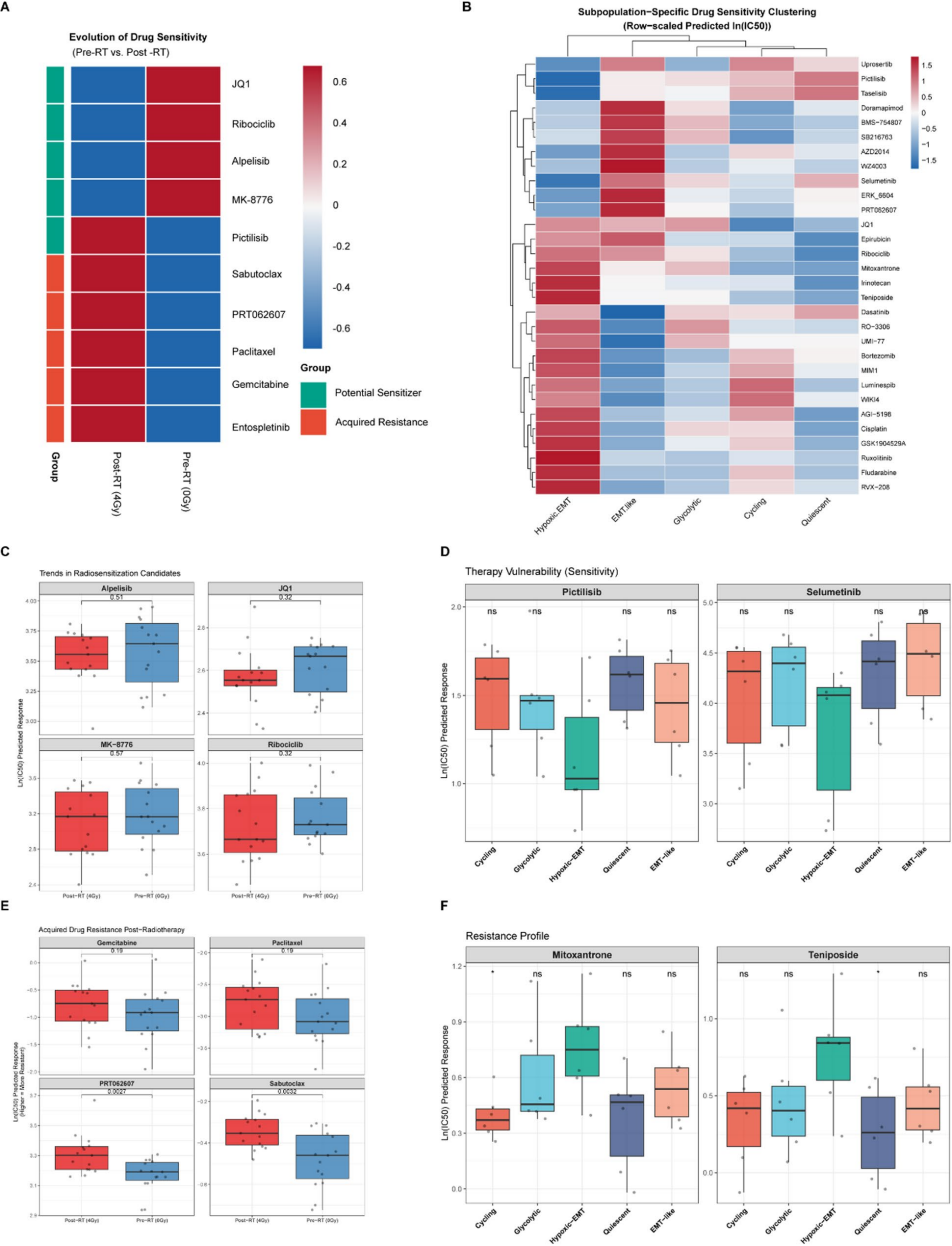
Prediction of drug sensitivity and identification of therapeutic targets. **A** Heatmap showing the differential drug response profiles between Pre-RT (0 Gy) and Post-RT (4 Gy) groups. Rows represent drugs, and columns represent groups. The color scale indicates the row-scaled predicted ln(IC50) values, where red represents higher IC50 (resistance) and blue represents lower IC50 (sensitivity). Drugs are categorized into “Potential Sensitizer” (Green bar) and “Acquired Resistance” (Orange bar) based on the shift in IC50 post-radiotherapy. **B** Hierarchical clustering heatmap of predicted drug sensitivities across the five identified cell subpopulations. The Hypoxic-EMT cluster (top rows) exhibits a distinct multidrug-resistance pattern compared to other clusters. **C** Box plots illustrating the predicted ln(IC50) values for top potential radiosensitizers (Alpelisib, JQ1, MK-8776, Ribociclib). The Post-RT group shows significantly lower ln(IC50) values, indicating increased sensitivity. Statistical significance was determined by the Wilcoxon rank-sum test. **D** Box plots showing the subpopulation-specific therapeutic vulnerabilities. The Hypoxic-EMT subpopulation exhibits specific sensitivity (lower IC50) to Pictilisib and Selumetinib compared to other clusters. **E** Box plots validating acquired resistance to standard chemotherapeutic agents (Gemcitabine, Paclitaxel, PRT06207, Sabutoclax) in the Post-RT group. **F** Box plots demonstrating the resistance profile of the Hypoxic-EMT subpopulation against Mitoxantrone and Teniposide, highlighting its role as a drug-resistant reservoir. (ns: not significant, *p < 0.05, **p < 0.01, ***p < 0.001)

To explore potential therapeutic strategies for overcoming radioresistance, we leveraged the oncopredict algorithm to estimate the half-maximal inhibitory concentration (IC50) of chemotherapeutic and targeted agents for single cells based on their transcriptomic profiles. Comparative analysis of drug sensitivity between Pre-RT (0 Gy) and Post-RT (4 Gy) groups revealed a distinct shift in the pharmacological landscape (Fig. 6A). As expected, organoids surviving radiotherapy exhibited acquired resistance to several broad-spectrum chemotherapeutic agents. Specifically, the predicted IC50 values for Gemcitabine and Paclitaxel were significantly elevated in the Post-RT group compared to untreated controls (Fig. 6E), suggesting that standard chemotherapy might be less effective for recurrent tumors.

However, this resistance was accompanied by emerging therapeutic vulnerabilities. We identified a subset of “Potential Sensitizers”—drugs to which the radioresistant cells became significantly more sensitive. Notably, the Post-RT group showed reduced IC50 values for JQ1 (BET inhibitor), Ribociclib (CDK4/6 inhibitor), and Alpelisib (PI3K inhibitor) (Fig. 6C). This suggests that targeting epigenetic regulators or cell cycle/PI3K pathways could be a viable strategy to eliminate residual radioresistant cells. We further dissected drug response heterogeneity at the subpopulation level (Fig. 6B). The Hypoxic-EMT subcluster emerged as a multidrug-resistant reservoir, exhibiting high predicted IC50 values for a broad range of compounds, including Mitoxantrone and Teniposide (Fig. 6F). In contrast, the Cycling and Glycolytic subpopulations appeared relatively more chemosensitive. Despite its general refractory nature, the Hypoxic-EMT subpopulation displayed specific vulnerability to Pictilisib (pan-PI3K inhibitor) and Selumetinib (MEK inhibitor) (Fig. 6D), indicating that targeting the *PI3K/MAPK* signaling axis might specifically ablate this resilient niche driving tumor recurrence.

## Discussion

Using PDO models integrated with single-cell transcriptomic profiling, this study systematically delineates the evolutionary landscape of NSCLC BM under radiotherapy pressure. It is worth emphasizing that our results consistently point to hypoxia as the core biological hub driving the formation of radiation tolerance. Radiotherapy not only exerts its killing effect by directly inducing DNA damage, but also exacerbates local oxygen deficiency through vascular damage and metabolic stress, thereby reshaping the tumor microenvironment [26, 27]. During this process, radiation-sensitive cells with high proliferation and high glycolysis are significantly eliminated, while cell subpopulations characterized by Hypoxic-EMT are selectively enriched, suggesting that hypoxia may be a key determinant of cell fate reprogramming after radiotherapy [28, 29].

The evolutionary trajectory further indicates that the activation of hypoxic signals occurs earlier than the emergence of the EMT phenotype, supporting its role as an upstream event in the process of drug resistance. Mechanistically, the microenvironmental pressure related to radiotherapy can trigger a transcriptional network centered on *HIF-1 A*, and by upregulating EMT regulatory factors such as *ZEB1*, it promotes the transformation of cells into a mesenchymal-like state [30, 31]. This “hypoxia-EMT” coupling not only reduces cell cycle activity but also enhances the cells’ tolerance to DNA damage and reactive oxygen species (ROS), allowing the tumor cells to enter a reversible low-proliferation survival state [32]. Thus, it is speculated that hypoxia not only shapes the resistant phenotype but may also provide a dynamic drug-tolerant persisters (DTPs) state for the residual cells, thereby laying the foundation for subsequent treatment recurrence [33, 34]. By acquiring this Hypoxic-EMT phenotype, tumor cells essentially establish a “resistant reservoir” capable of evading acute cytotoxic stress and fueling subsequent relapse.

Mechanistically, we identified the NF-κB/STAT-driven “viral mimicry” response as a central hub of this adaptive network [35, 36]. Radiation-induced cytoplasmic DNA accumulation typically triggers *cGAS-STING* sensing to activate type I interferon signaling [37–39]. While this “viral mimicry” is often harnessed to prime anti-tumor immunity, our data reveals its distinct cell-intrinsic pro-survival role in the absence of immune effectors. Instead of triggering immune clearance, the activated NF-κB and STAT signals paradoxically upregulate anti-apoptotic effectors (e.g., *XIAP*) [26, 40]. This highlights a “double-edged sword” nature of radiation-induced inflammation, where intrinsic stress adaptation mechanisms override canonical immune-stimulatory signals to promote tumor survival.

During the process of transcriptional reprogramming, this study further revealed that the lung cancer brain metastasis cells underwent a profound reconfiguration towards a metabolically flexible state under radiotherapy stress. Previous studies have shown that tumor hypoxia is a common feature of solid tumors, which can drive the formation of drug resistance by promoting tumor cell survival, enhancing invasiveness, and weakening treatment response [41]. In this context, we observed that the hypoxic-EMT subpopulation with radioresistance did not rely on a single energy pathway but simultaneously maintained multiple energy supply pathways such as glycolysis, mitochondrial oxidative phosphorylation, and fatty acid oxidation, thereby meeting the high biological energy requirements for damage repair and long-term survival. Existing research has also pointed that fatty acid oxidation can inhibit cell apoptosis by enhancing mitochondrial membrane stability and is an important metabolic support for treatment-resistant tumors [42]. At the same time, the EMT process itself is accompanied by metabolic reprogramming and is considered a potential intervention target for improving tumor treatment response and prognosis [43]. Moreover, enhanced glutathione metabolism further enhanced the cell’s ability to clear reactive oxygen species, enabling it to resist radiotherapy-induced oxidative damage and maintain a reversible low-proliferation survival state [44]. In summary, this metabolic adaptation driven by hypoxia not only explains the persistence of residual cells after radiotherapy but also suggests that targeting glycolysis alone may not be sufficient to overcome drug resistance, and combined inhibition of key metabolic dependencies such as oxidative phosphorylation or fatty acid oxidation may become a more promising therapeutic strategy for enhancing the radiosensitivity of LCBM.

Importantly, our study translates these molecular insights into actionable pharmacological strategies. While the post-radiotherapy landscape exhibited cross-resistance to conventional chemotherapeutics like paclitaxel and gemcitabine [45]. It exposed distinct “collateral vulnerabilities.” We found that the radioresistant Hypoxic-EMT subpopulation is hypersensitive to the inhibition of the *PI3K/MAPK* signaling axis [46]. This suggests that sequential or combinatorial regimens integrating radiotherapy with agents such as Alpelisib (PI3K inhibitor) or Selumetinib (MEK inhibitor) could selectively ablate the radiation-tolerant reservoir, thereby preventing the reseeding of recurrent tumors [47, 48].

Despite the comprehensive insights provided by our single-cell atlas, several limitations of this study warrant consideration. First, while patient-derived organoids faithfully recapitulate the genomic and histological features of parental tumors, they inherently lack the complex tumor microenvironment (TME), particularly immune and stromal components. Consequently, our study focused on the tumor-intrinsic adaptive response to radiation-induced “viral mimicry” signals. We could not evaluate how these signals might orchestrate immune cell recruitment or activation in an immunocompetent setting, which is a critical aspect of radiotherapy efficacy in vivo. Second, although the use of paired pre- and post-treatment samples minimizes background noise, the sample size in this study was relatively limited. Given the high inter-patient heterogeneity of LCBM, future studies with larger cohorts are needed to validate the universality of the Hypoxic-EMT trajectory and the identified metabolic targets. Third, our findings regarding metabolic reprogramming and drug vulnerabilities are primarily based on transcriptomic inference. While computational approaches such as scMetabolism and oncoPredict provide valuable predictive insights, it should be noted that the oncoPredict model is trained on bulk cancer cell line datasets. As such, its application to single-cell transcriptomic data may introduce potential bias. In addition, post-transcriptional modifications and enzymatic activities can dissociate gene expression from actual metabolic flux. Therefore, further functional validation using metabolomics, stable isotope tracing, and in vivo efficacy assays in PDX models is essential to translate these findings into clinical practice.

In conclusion, our results outline a multidimensional roadmap for overcoming radioresistance in LCBM. While our PDO model focuses on tumor-intrinsic adaptations, future studies incorporating immune components are warranted to elucidate the interplay between radiation-induced viral mimicry and the tumor microenvironment [49, 50]. Nevertheless, the identification of the Hypoxic-EMT state, its metabolic dependencies (FAO/OXPHOS), and its specific drug sensitivities provides a robust framework for designing precision combination therapies [51, 52]. By monitoring subpopulation dynamics and targeting these acquired vulnerabilities, we may significantly improve the prognosis for patients with brain metastases.

## Conclusion

Our study delineates a multidimensional adaptive network wherein LCBM cells achieve radiotherapy tolerance through coordinated transcriptional reprogramming, metabolic switching, and phenotypic plasticity toward a Hypoxic-EMT state. Targeting the uncovered NF-κB-mediated viral mimicry responses or specific metabolic dependencies of these resistant subpopulations represents a promising therapeutic avenue. Ultimately, our findings suggest that exploiting the collateral sensitivity to PI3K/MAPK inhibitors offers a viable strategy to overcome radiotherapy resistance in lung cancer brain metastasis.

## Data Availability

The datasets used and/or analysed during the current study are available from the corresponding author on reasonable request.
